# Knowledge About and the Use of Oral Nonsteroidal Anti-inflammatory Drugs Among Patients With Rheumatic Disorders in Saudi Arabia: A Cross-Sectional Study

**DOI:** 10.7759/cureus.48500

**Published:** 2023-11-08

**Authors:** Ashwaq O Alharbi, Sarah A Almjayishi, Rahaf I Aldarwish, Manal A Almeshari, Meshari T Aljumydi, Rami M Faraj

**Affiliations:** 1 College of Pharmacy, Qassim University, Buraydah, SAU; 2 College of Pharmacy, King Abdulaziz University, Jeddah, SAU; 3 College of Pharmacy, King Khalid University, Abha, SAU; 4 College of Pharmacy, Prince Sattam Bin Abdulaziz University, Al Kharj, SAU; 5 College of Pharmacy, Ibn Sina National College for Medical Studies, Jeddah, SAU

**Keywords:** (nsaids) nonsteroidal anti-inflammatory drugs, (nsaid) nonsteroidal anti-inflammatory drugs, oral nonsteroidal anti-inflammatory drugs, saudi arabia, rheumatic disorders, side effects, patient’s knowledge

## Abstract

Aims: The aims were to evaluate rheumatic disorder patients' knowledge of the side effects of nonsteroidal anti-inflammatory drugs (NSAIDs), dosage (from where the knowledge is acquired), contraindications (in pregnancy), and drug interactions (such as drug-related disorders), to improve their knowledge about taking NSAIDs in Saudi Arabia.

Study design: It is a descriptive cross-sectional study.

Place and duration of study: The study was conducted among patients with rheumatic disorder (RD) in Saudi Arabia who were invited to join by completing an online questionnaire, from August to November 2022.

Methodology: Patients with RD who were 16 years and older, received oral NSAIDs, and agreed to participate were included in the study. The data was collected using an Internet-based questionnaire. The Internet-based questionnaire was designed using the Google Forms (Google LLC,* *Mountain View, California, United States) online survey platform. A consent form accompanied the questionnaire in a cover letter briefly describing the investigation.

Results: A total of 756 participants answered the questionnaire and participated in the final analysis. Most of the participants (75.93%) were female. The type of NSAID used by the participants was Ibuprofen. The majority (83.41%) reported that they responded to the medication. When asked about the primary source of information about the correct dose of these medications, more than two-thirds (69.32%) reported that physicians are the main source of information. The majority of the participants (83.39%) denied using NSAIDs during pregnancy. Half of the participants (49.22%) said they had discussed these medications’ harmful effects with their doctors or pharmacists. The female participants were more knowledgeable about NSAIDs and their side effects than the males.

Conclusion: The patient's knowledge related to the use of oral NSAIDs was not that satisfactory. There is an urgent need to educate the public regarding the appropriate use of NSAIDs by healthcare providers, particularly in relation to their proper use, potential side effects, and risks associated with long-term use.

## Introduction

Nonsteroidal anti-inflammatory drugs (NSAIDs) are the most widely used analgesics with a range of therapeutic applications for both over-the-counter (OTC) and prescription-based variants. They accounted for 67% of analgesic use in Saudi Arabia between 2010 and 2015, ranking among the top 10 most popular medications overall [1].

NSAIDs work by suppressing the cyclooxygenase (COX) enzyme, which converts arachidonic acid into thromboxanes, prostaglandins, and prostacyclins. The lack of these eicosanoids contributes to the therapeutic effects of NSAIDs. However, COX-1 suppression can prevent the production of protective prostaglandins in the gastric mucosa, leading to adverse gastrointestinal effects [2], such as erosions and ulcers, with consequences including bleeding, protein loss, the development of strictures, and perforation [3,4]. Despite the use of proton pump inhibitors, the celecoxib group experienced significantly fewer gastric adverse events than the naproxen or ibuprofen groups [5]. Prostaglandins also play a crucial role in regulating renal hemodynamics. In a patient with normal renal function, inhibiting prostaglandin synthesis may not have significant consequences. However, in a patient with renal impairment, these prostaglandins play a more critical role and can lead to complications when reduced by NSAIDs.

Complications such as acute renal dysfunction, fluid and electrolyte imbalances, renal papillary necrosis, and nephrotic syndrome/interstitial nephritis can occur [2]. Additionally, there is a kidney-heart connection; by decreasing renal COX-2, NSAIDs can increase the levels of asymmetric dimethyl arginine (ADMA), which is known to inhibit the production of nitric oxide (NO). Reducing NO levels in the kidneys can have several effects, leading to an increase in hypertension, atherosclerosis, and thrombosis [6]. 

Pharmacokinetics and pharmacodynamics can also contribute to adverse cardiac effects from NSAID use. The strength and plasma half-life of the NSAID can affect the ratio of COX-1 to COX-2 inhibition during the medication's dosing interval [2]. For example, during the dosing interval for diclofenac, COX -1 inhibition declines as the plasma concentration decreases, leaving COX-2 inhibition largely uncontrolled. In contrast, COX-1 inhibition is greater than COX-2 inhibition for both ibuprofen and naproxen across the dosage interval [2]. Therefore, the choice of NSAID depends on the patient's risk factors, medical history, and other medications. Patients with a high risk of GI bleeding or those taking anticoagulants may benefit from a selective COX-2 inhibitor or a weaker NSAID. Patients with cardiovascular disease or risk factors may benefit from taking a low dose of aspirin, which selectively inhibits COX-1 and reduces the risk of thrombosis.

Patients with rheumatic disorders (RDs) often require knowledge, depending on their case. However, research shows that patients do not usually know the information needed about NSAIDs [1].

This literature review examines the knowledge and awareness of NSAIDs among patients and pharmacy customers. Several studies have investigated the relationship between factors such as education, income, and understanding of the side effects of NSAIDs [7-9].

Overall, the findings suggest that as time passes, knowledge of NSAID drug interactions, side effects, and dosage instructions decreases, particularly among those with lower levels of education [7-9]. Patients may not receive updated information about their medications from healthcare providers regularly. In addition, patients with lower levels of education may have more difficulty understanding complex medical information, making it harder for them to retain and remember instructions over time. These findings confirm the importance of ongoing patient education and communication between patients and their healthcare providers.

A study conducted by Arain et al. in 2019 found that patients with higher income levels had greater awareness of the side effects of OTC NSAIDs compared to patients with lower incomes. Specifically, patients in the higher income group were more likely to correctly identify specific side effects of OTC NSAIDs. They were more likely to report receiving information about these side effects from a healthcare provider. They may also have greater access to healthcare information and resources and be more likely to see a healthcare provider regularly [7]. Healthcare providers should be aware of the possible differences in patient awareness and understanding of OTC NSAID side effects based on income level. They should prioritize providing clear and comprehensive information to all patients, regardless of income.

More than half of the orthopedic patients taking NSAIDs for at least one month expressed concern that knowledge about adverse drug reactions (ADRs) may lead them to discontinue their prescribed medications in a study done at King Khalid University Hospital in Riyadh, Saudi Arabia [10]. Pharmacists and physicians are responsible for fully educating patients about ADRs and clarifying their roles in helping prevent these reactions to avoid negative impacts on adherence.

Patients or pharmacy customers have reported the high frequency of adverse effects, lack of safety usage, and inadequate knowledge of NSAID adverse effects and drug interactions [10].

On the other hand, a study involving patients who visited a rheumatology clinic taking OTC NSAIDs exposed them to a list of adverse effects, including indigestion, headache, dizziness, drowsiness, fluid retention, kidney problems, liver problems, and heart/circulation issues [8]. Most patients reported being aware of at least three side effects [8]. However, only 40.5% were aware of their medication's adverse effects in data gathered among Saudi Arabians [9].

In summary, recent studies suggest that the general public still has limited knowledge of NSAIDs, including adverse effects and drug interactions [7-9]. Given that adverse drug reactions to NSAIDs are a leading cause of hospital admissions, there is a need for more outstanding education and awareness-raising campaigns to help patients and pharmacy customers better understand the risks associated with these drugs [9,10].

The study's objective was to assess the knowledge and awareness of rheumatology patients in Saudi Arabia regarding oral NSAIDs, which are crucial for managing autoimmune and auto-inflammatory disorders. Patients who lack knowledge of NSAIDs may have a higher risk of adverse effects, a worse prognosis, and increased healthcare costs, due to comorbidities and concurrent medications. This study aims to evaluate patients' understanding of NSAIDs' side effects, dosage(from where they acquire the knowledge), contraindications (in pregnancy), and drug interactions (such as drug-related disorders) to improve their knowledge about NSAIDs. With the help of healthcare providers, we improve patients' awareness of NSAIDs by informing them of the appropriate dosage, side effects, and contraindications.

## Materials and methods

Following approval from the National Committee of Bio-Ethics at Qassim region, Saudi Arabia, with registration number 607-45-2043, a descriptive cross-sectional study was conducted among patients with RD in Saudi Arabia who were invited to join by completing an online questionnaire from August to November 2022. Patients who were 16 years and older, received oral NSAIDs, and agreed to participate were included in the study. Patients without RD, who were less than 16 years old, had not received any oral NSAIDs, and refused to cooperate with us were excluded.

The data were collected using an Internet-based questionnaire. The Internet-based questionnaire was designed using the Google Forms (Google LLC, Mountain View, California, United States) online survey platform. A consent form accompanied the questionnaire in a cover letter briefly describing the investigation.

A questionnaire comprising two sections was used. The first section involved demographic information such as age, gender, education levels, and income. The second section included questions assessing patients’ knowledge of NSAIDs and contained four parts: diagnosis, administration, side effects and contraindications, and cost. The question-and-answer types varied from open-ended to closed-ended questions. The original questionnaire items were available in English; however, the material was translated into Arabic [8]. The survey was piloted among a few patients for validity. The survey was promoted through possible data collection strategies (i.e., smartphone applications such as Telegram (Telegram Messenger Inc., Tortola, British Virgin Islands)). Appendix: A contains detailed information on the questionnaire.

IBM SPSS Statistics for Windows, Version 26.0 (Released 2019; IBM Corp., Armonk, New York, United States) was used to analyze the data. For each question, descriptive statistics, such as frequency and percentage, were generated. To test the significance of the gender, we used an independent t-test and one-way analysis of variance (ANOVA) for the educational level.

## Results

The demographic characteristics of the participants (N = 756) are presented in Table 1. The gender distribution revealed that 75.93% of the participants identified as female, while 24.07% identified as male. In terms of education level, the majority of participants held a bachelor's degree or higher (73.81%), with smaller proportions having completed high school (18.65%), secondary school (3.70%), or elementary school (2.12%). A negligible percentage fell into the "Others" category (1.72%). When considering income levels, the data indicated that 47.62% of participants reported earning between 1000 to 3000 Saudi Arabian Riyal (SAR), 11.11% earned between 3000 to 5000 SAR, 14.02% earned between 5000 to 7000 SAR, and 27.25% earned more than 7000 SAR. The participants' ages ranged from 18 to 65 years, with a mean age of 32.88 years (SD = 10.75).

**Table 1:** The demographic characteristics of the participants (N = 756). SAR: Saudi Arabian Riyal

Characteristics	Subgroup	Frequency	%
Gender	Female	574	75.93%
	Male	182	24.07%
Education level	Elementary school	16	2.12%
	Secondary school	28	3.70%
	High school	141	18.65%
	Bachelor and above	558	73.81%
	Others	13	1.72%
Income level	1000-3000 SAR	360	47.62%
	3000-5000 SAR	84	11.11%
	5000-7000 SAR	106	14.02%
	More than 7000 SAR	206	27.25%
Age (years)	Mean	SD	
	32.88	10.75	

Table 2 outlines the participants' usage of anti-inflammatory painkillers. Regarding changes in anti-inflammatory painkillers, 29.64% (n = 171) of participants reported having made changes, while the majority (70.36%, n = 406) had not. Similarly, 24.44% (n = 141) indicated changing the drug company producing their medication, while 75.56% (n = 436) had not. Approximately 57.37% (n = 331) of respondents reported receiving painkillers for free, whereas 42.63% (n = 246) did not. Of the participants, 49.22% (n = 284) stated that they had discussed the adverse effects of NSAIDs with a doctor or pharmacist, while 50.78% (n = 293) had not engaged in such discussions. Moreover, 21.84% (n = 126) of participants experienced side effects from the painkillers, while the majority (78.16%, n = 451) did not encounter any adverse effects. For those who did not experience side effects, the mean cost of purchasing these painkillers per month was 122.65 SAR, with SD of 180.41.

**Table 2:** Using anti-inflammatory painkillers among the participants. NSAIDs: nonsteroidal anti-inflammatory drugs; SAR: Saudi Arabian Riyal

Question	Response	Frequency	%
Have you changed your anti-inflammatory painkillers?	No	406	70.36%
	Yes	171	29.64%
Have you ever changed the drug company?	No	436	75.56%
	Yes	141	24.44%
Do you get these painkillers for free?	No	246	42.63%
	Yes	331	57.37%
Have you ever discussed with your doctor or pharmacist the adverse effects of NSAIDs?	No	293	50.78%
	Yes	284	49.22%
Have you had any side effects from these painkillers?	No	451	78.16%
	Yes	126	21.84%
If the answer is no, mention how much it costs you (SAR) to buy it per month.	Mean	SD	
	122.65	180.41	

In Table 3, participants' usage of specific NSAIDs is detailed. Among the surveyed participants (N = 756), the frequency of using various NSAIDs was as follows: ibuprofen (11.38%), naproxen (7.14%), celecoxib (11.38%), aspirin > 100 mg (5.03%), diclofenac (22.75%), meloxicam (7.67%), etoricoxib (5.69%), and other NSAIDs (5.29%). Notably, 23.68% of participants indicated not using any of the listed NSAIDs.

**Table 3:** NSAIDs used by the participants. NSAIDs: nonsteroidal anti-inflammatory drugs

Question	Response	Frequency	%
Have you ever used any of the following medicines? (N = 756)	Ibuprofen	86	11.38%
	Naproxen	54	7.14%
	Celecoxib	86	11.38%
	Aspirin > 100 mg	38	5.03%
	Diclofenac	172	22.75%
	Meloxicam	58	7.67%
	Etoricoxib	43	5.69%
	Others	40	5.29%
	None	179	23.68%

**Figure 1:**
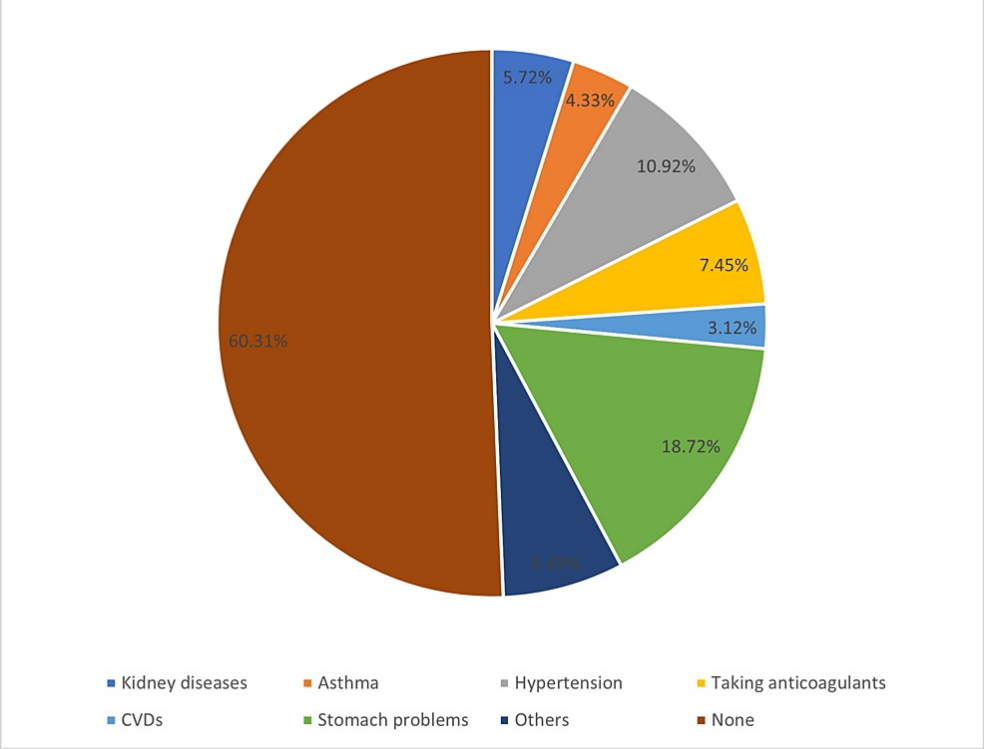
Comorbidities information of the participants.

Table 4 and Figure 1 provide insights into the relationship between NSAID use and the presence of comorbidities among participants. Concerning NSAID usage during pregnancy, 16.61% (n = 49) of participants indicated using oral NSAIDs, with 95.92% (n = 47) of these individuals taking them with a prescription. Among respondents, 77.64% (n = 448) reported having read the instructions or cautions on drug leaflets, and of these, 42.82% (n = 176) had at some point discontinued use due to the concerns they read in the leaflets. Regarding specific comorbidities, 5.72% (n = 33) of participants reported suffering from kidney diseases, 4.33% (n = 25) had asthma, 10.92% (n = 63) had hypertension, 7.45% (n = 43) were taking anticoagulants, 3.12% (n = 18) had cardiovascular diseases, 18.72% (n = 108) had stomach problems, and 8.49% (n = 49) indicated other health issues. Conversely, the majority of participants did not suffer from these specific conditions. Notably, 60.31% (n = 348) reported having no comorbidities.

**Table 4:** Use of NSAIDs and the presence of comorbidities among the participants. CVDs: cardiovascular diseases; none: do not suffer from any; other: other than the diseases mentioned above

Question	Response	Frequency	%
During pregnancy, did you use oral NSAID?	No	246	83.39%
	Yes	49	16.61%
If the answer is yes, did you take it with a prescription?	No	2	4.08%
	Yes	47	95.92%
Have you ever read the instructions or cautions on the drug leaflet?	No	129	22.36%
	Yes	448	77.64%
If the answer is yes, have you ever stopped taking it because of your fear of what was mentioned in the leaflet?	No	235	57.18%
	Yes	176	42.82%
Do you suffer from any of the following? (kidney diseases)	No	544	94.28%
	Yes	33	5.72%
Do you suffer from any of the following? (asthma)	No	552	95.67%
	Yes	25	4.33%
Do you suffer from any of the following? (hypertension)	No	514	89.08%
	Yes	63	10.92%
Do you suffer from any of the following? (taking anticoagulants)	No	534	92.55%
	Yes	43	7.45%
Do you suffer from any of the following? (CVDs)	No	559	96.88%
	Yes	18	3.12%
Do you suffer from any of the following? (stomach problems)	No	469	81.28%
	Yes	108	18.72%
Do you suffer from any of the following? (none)	No	229	39.69%
	Yes	348	60.31%
Do you suffer from any of the following? (others)	No	528	91.51%
	Yes	49	8.49%

Table 5 illustrates participants' responses concerning the effectiveness of NSAIDs and the sources from which they obtain information about the correct dose. Among those who had used the mentioned NSAIDs (N = 440), 83.41% (n = 367) reported a positive response to the medications, while 16.59% (n = 73) did not. Regarding information sources for correct NSAID dosing (N = 577), physicians were the primary source for 69.32% (n = 400) of participants, followed by pharmacists (16.98%, n = 98), taking it as needed (10.05%, n = 58), leaflet instructions (2.77%, n = 16), and internet/magazines (0.87%, n = 5).

**Table 5:** Response of participants to NSAIDs and source of information regarding correct dose. ‡Regardless of the correct dose. NSAIDs: nonsteroidal anti-inflammatory drugs

Question	Response	Frequency	%
If you have used any of the above-mentioned NSAIDs, did you respond to it? (N = 440)	No	73	16.59%
	Yes	367	83.41%
From where did you get the information about the correct dose of NSAIDs? (N = 577)	Pharmacist	98	16.98%
	Physician	400	69.32%
	Leaflet instructions	16	2.77%
	Take it as needed^‡^	58	10.05%
	Internet/magazine	5	0.87%

Table 6 presents participants' knowledge about NSAIDs and their side effects. Results indicate that 50.61% (n = 292) were aware that oral NSAIDs might induce gastrointestinal issues, 33.10% (n = 191) recognized the potential for renal issues, 39.34% (n = 227) knew about the possibility of increased blood pressure, 35.88% (n = 207) were aware of the risk of heart attack and stroke, and 54.25% (n = 313) knew that oral NSAIDs should be taken with food.

**Table 6:** Knowledge about NSAIDs and their side effects. NSAIDs: nonsteroidal anti-inflammatory drugs

Question	Response	Frequency	%
Do you know that oral NSAIDs might induce gastrointestinal issues such as heartburn and stomach ulcer?	No	285	49.39%
	Yes	292	50.61%
Do you know that oral NSAIDs can cause renal issues, including kidney failure?	No	386	66.90%
	Yes	191	33.10%
Do you know that oral NSAIDs can raise blood pressure?	No	350	60.66%
	Yes	227	39.34%
Do you know that oral NSAIDs can increase the risk of heart attack and stroke?	No	370	64.12%
	Yes	207	35.88%
Do you know that oral NSAIDs should be taken with food?	No	264	45.75%
	Yes	313	54.25%

Table 7 explores factors influencing participants' knowledge levels about NSAIDs and their side effects. Significant differences were observed based on gender (P = 0.011), discussions with doctors or pharmacists (P = 0.099), reading leaflet instructions (P = 0.077), and the source of information about correct NSAID dosing (P < 0.001). Education level and income level were not found to have significant impacts on knowledge scores. 

**Table 7:** Factors affecting the level of knowledge about NSAIDs and their side effects. ‡Regardless of the correct dose. NSAIDs: nonsteroidal anti-inflammatory drugs; SAR: Saudi Arabian Riyal

Factors	Subgroup/Response	Knowledge Score		
		Mean	SD	P-Value
Gender	Female	1.72	1.74	0.011
	Male	1.35	1.62	
Education level	Elementary school	1.31	0.87	0.158
	Secondary school	1.11	1.23	
	High school	1.42	1.67	
	Bachelor and above	1.71	1.77	
	Others	1.69	1.6	
Income level	1000-3000 SAR	1.52	1.7	0.180
	3000-5000 SAR	1.48	1.75	
	5000-7000 SAR	1.71	1.59	
	> 7000 SAR	1.83	1.78	
Discussions with doctors or pharmacists	No	1.92	1.65	0.099
	Yes	2.35	1.66	
Reading the instructions or cautions on the drug leaflet?	No	1.15	1.43	0.077
	Yes	2.42	1.63	
The source of information about the correct dose of NSAIDs?	Pharmacist	2.57	1.78	< 0.001
	Physician	1.93	1.62	
	The leaflet instructions	2.88	1.45	
	Take it as needed^‡^	2.43	1.69	
	Internet/magazine	3.4	1.14	

## Discussion

The results provided valuable insights into the patient’s demographic characteristics, patterns of NSAIDs use, and knowledge about NSAIDs and their side effects. Most of the participants in this study were females, which is consistent with the higher prevalence of RD among women. The average age of the participants was relatively young, indicating that rheumatic diseases affect individuals at a relatively early age in Saudi Arabia. Furthermore, most of the participants had a bachelor’s degree or higher education level, suggesting that they may have a higher level of health literacy and understanding of their condition and medications.

The findings regarding participants' usage of NSAIDs reveal interesting trends. A significant proportion of participants reported changes in their NSAID usage and drug company preference, indicating potential concerns or preferences for specific brands or formulations. The prevalence of participants receiving painkillers for free may reflect the healthcare system’s support in Saudi Arabia [11]. A considerable number of participants reported discussing adverse effects with healthcare professionals, emphasizing the importance of patient-provider communication in managing medication-related concerns. The findings suggest that participants’ NSAID usage is influenced by factors such as concerns or preferences for certain brands or formulations [12]. The shift in drug company preference implies the role of marketing strategies and perceived effectiveness.

The usage pattern of specific NSAIDs among participants sheds light on their preferences and potentially perceived effectiveness of these medications. Notably, a substantial percentage of participants did not use any of the listed NSAIDs, possibly indicating alternative pain management strategies or variations in the severity of their conditions. The information on comorbidities and NSAIDs used during pregnancy provides crucial insights into the complex decision-making processes patients face when managing multiple health concerns. Studies indicated that individuals often choose NSAIDs based on perceived effectiveness and personal preferences. However, the percentage of non-NSAID users and factors influencing these decisions may vary based on cultural and demographic differences [13,14].

The positive response reported by the majority of participants regarding NSAID effectiveness underscores the importance of these medications in alleviating symptoms among patients with RD [15]. The study’s findings regarding knowledge levels about NSAID side effects reveal a mixed understanding among participants. While some participants were aware of potential risks associated with NSAID use, others demonstrated gaps in their knowledge. This variance in knowledge underscores the importance of tailored educational interventions aimed at enhancing patients’ awareness of the potential side effects and risks of NSAID use. Studies have shown that targeted interventions, such as informational pamphlets and counseling sessions, can effectively bridge the knowledge gaps and empower patients to make informed decisions about NSAID use [10, 16].

The identification of factors influencing participants' knowledge levels about NSAIDs and their side effects provides valuable insights for healthcare practitioners and policymakers. The observed significant differences are based on discussions with healthcare professionals and information sources. This suggests that open and informative conversations between patients and healthcare providers play a key role in enhancing patients’ knowledge about NSAIDs and their potential side effects [17]. Additionally, the importance of reliable sources of information cannot be understated, as these sources contribute significantly to patients’ comprehension of medication-related information. Prior research shows that a consistent trend emerges, where patient education from healthcare providers and access to credible information sources are influential in shaping patients’ knowledge about medications [18].

The study's findings have important implications for healthcare professionals, policymakers, and patient education initiatives. Based on the results, strategies could be developed to enhance patient-provider communication, ensuring that patients receive comprehensive information about the benefits and risks of NSAID usage. Targeted educational programs could address the gaps in knowledge observed among participants, particularly concerning the potential side effects of NSAIDs. Additionally, healthcare providers can play a pivotal role in guiding patients toward reliable information sources for correct NSAID dosing [19,20].

It's important to acknowledge the limitations of the study, such as the cross-sectional design, which restricts the establishment of causal relationships. Further research could employ longitudinal designs to explore changes in NSAID usage patterns and knowledge levels over time. Additionally, the study’s focus on a specific population in Saudi Arabia might limit the generalizability of the findings to broader populations or cultural contexts.

## Conclusions

This study provides important insights into the knowledge and use of oral NSAIDs among patients with RD in Saudi Arabia. The patient's knowledge of oral NSAID use was not that satisfactory. The findings highlight the need for healthcare providers to improve patient education about NSAIDs, particularly regarding their appropriate use, potential side effects, and risks associated with long-term use.

## Appendices

Questionnaire

The purpose of this study was to evaluate the knowledge of rheumatic patients about the dosage, side effects, contraindications, and drug interactions of oral NSAIDs.

Summary

The main research question of the study was to evaluate the knowledge and utilization of oral NSAIDs among patients with rheumatic disorders in Saudi Arabia. The study found that patients' knowledge of oral NSAID use was insufficient, indicating the need for improved patient education regarding NSAIDs. The study highlights the importance of providing patients with information about proper NSAID usage, potential side effects, and long-term risks.

In terms of fitting within the existing literature, this study adds to a growing body of evidence that highlights the limited knowledge among patients regarding NSAID usage. It specifically focuses on patients with rheumatic disorders in the context of Saudi Arabia, contributing to the literature in that specific region.

The conclusion of the study underscores the necessity for healthcare providers to prioritize patient education campaigns and interventions to address the knowledge gap and ensure that patients have a comprehensive understanding of the benefits, risks, and proper usage of oral NSAIDs.

Assessment

1. Introduction: The introduction provides a clear overview of the importance of reliable sources of information and their impact on patients' comprehension of medication-related information. It effectively sets the stage for the study and highlights the gaps in knowledge that the research aims to address.

2. Method: The methodology section provides sufficient information about the study design, sample, and data collection methods. However, one potential weakness is the cross-sectional design, which limits the ability to establish causal relationships between variables. A longitudinal design would have been beneficial to explore changes in NSAID usage patterns and knowledge levels over time.

3. Results: The results are presented clearly, with relevant statistical measures and descriptive analyses. However, it would be helpful to provide more contextual information about the significance of the findings and how they contribute to the existing literature.

4. Discussion: The discussion provides a comprehensive analysis of the study's findings and their implications. It effectively highlights the importance of healthcare professionals, policymakers, and patient education initiatives in improving patient-provider communication and addressing knowledge gaps. The limitations of the study, such as the cross-sectional design and the specific population focus, are acknowledged.

5. Conclusions: The conclusions drawn from the study are consistent with the evidence presented. The study's findings emphasize the need for strategies to enhance patient-provider communication, targeted educational programs, and the role of healthcare providers in guiding patients to reliable information sources.

In terms of areas of weakness or flaws, as previously mentioned, the cross-sectional design limits the ability to establish causal relationships. Additionally, the study's focus on a specific population in Saudi Arabia may limit the generalizability of the findings to broader populations or cultural contexts.

Target group: rheumatic patients

Section I: Demographic Questions

We hope that you, as the study sample and its focus, will answer the questionnaire questions, knowing that your answers will be treated in strict confidence and will be used for scientific research purposes only.

Participation is only for those aged 16 years and above.

Do you agree to participate?

●      Yes

●      No

**Table 8:** Section I: Demographic Questions

	Age:	
	Gender:	Female Male
	Education level:	Elementary Intermediate Secondary Bachelors Other: ..................
	Income level:	1,000-3,000 SAR 3000-5000 SAR 5,000-7,000 SAR Above 7000.

Section II: Assessment of the Extent of Knowledge About Oral NSAIDs

**Table 9:** Part I: Diagnosis

	Have you ever used any of the following medications:	Ibuprofen, Naproxen, Celecoxib, Aspirin (more than 100 mg), Diclofenac, Meloxicam, Etoricoxib, I don't use any of these medications, Others: ..................
	Do you have a clear diagnosis that you should use it for? State the diagnosis.	Yes, there is a diagnosis: ............. There is no diagnosis.
	If the answer to the previous question is yes, do you respond to analgesics?	Yes No

**Table 10:** Part II: Administration

	What is the dose of this analgesic? How many times per day do you take? And for how long?	
	From where do you get the correct dose information for these medications:	Pharmacist, Physician, See the instructions in the drug leaflet, Take it as needed, regardless of dose, From the Internet or from the magazine, Others: ..............
	During pregnancy, did you use oral NSAIDs?	Yes No
	If the answer to the previous question is yes, would you take it with a prescription?	Yes No
	Have you ever read the instructions or warnings in the drug leaflet?	Yes No
	If the answer to the previous question is yes, have you ever stopped taking it because of your fear of what was mentioned in the leaflet?	Yes No

**Table 11:** Part III: Side Effects and Contraindications

	Do you suffer from any of the following:	Kidney problems (such as chronic kidney disease). Bronchial asthma, High blood pressure, Taking any blood thinners, Heart disease (e.g., angina, coronary artery disease, chest pain during exercise), Stomach problem (e.g., peptic ulcer disease, gastritis, or previous gastrointestinal bleeding). Others: .................. Do not suffer from any.
	Have you discussed with your doctor or pharmacist about the side effects of these medications?	Yes No
	Have you had any side effects from these analgesics?	Yes No
	If the answer to the previous question is yes, mention the side effect and how it was treated.	Yes No
	Do oral NSAIDs induce gastrointestinal problems (such as heartburn and stomach ulcers)?	Yes No
	Do oral NSAIDs cause kidney problems, including kidney failure?	Yes No
	Do oral NSAIDs raise blood pressure?	Yes No
	Do oral NSAIDs increase the risk of heart attacks and strokes?	Yes No
	Should oral NSAIDs be taken with food?	Yes No

**Table 12:** Part IV: Cost

Yes No	Have your anti-inflammatory analgesics been changed?	
	If the answer to the previous question is yes, mention how often the analgesic was changed? And how long was the duration between them?	
Yes No	Have you ever changed the drug company?	
	If yes, mention the first and second companies.	
Yes No	Do you get these analgesics for free?	
	If the answer to the previous question is no, indicate how much it costs to buy it per month.	
